# Exploring Novel Molecular Targets for the Treatment of High-Grade Astrocytomas Using Peptide Therapeutics: An Overview

**DOI:** 10.3390/cells9020490

**Published:** 2020-02-20

**Authors:** Giulia Guidotti, Liliana Brambilla, Daniela Rossi

**Affiliations:** Istituti Clinici Scientifici Maugeri IRCCS, Laboratory for Research on Neurodegenerative Disorders, 27100 Pavia, Italy; giulia.guidotti@icsmaugeri.it (G.G.); liliana.brambilla@icsmaugeri.it (L.B.)

**Keywords:** astrocytomas, peptide-based therapy, molecular targets, rehabilitation

## Abstract

Diffuse astrocytomas are the most aggressive and lethal glial tumors of the central nervous system (CNS). Their high cellular heterogeneity and the presence of specific barriers, i.e., blood–brain barrier (BBB) and tumor barrier, make these cancers poorly responsive to all kinds of currently available therapies. Standard therapeutic approaches developed to prevent astrocytoma progression, such as chemotherapy and radiotherapy, do not improve the average survival of patients. However, the recent identification of key genetic alterations and molecular signatures specific for astrocytomas has allowed the advent of novel targeted therapies, potentially more efficient and characterized by fewer side effects. Among others, peptides have emerged as promising therapeutic agents, due to their numerous advantages when compared to standard chemotherapeutics. They can be employed as (i) pharmacologically active agents, which promote the reduction of tumor growth; or (ii) carriers, either to facilitate the translocation of drugs through brain, tumor, and cellular barriers, or to target tumor-specific receptors. Since several pathways are normally altered in malignant gliomas, better outcomes may result from combining multi-target strategies rather than targeting a single effector. In the last years, several preclinical studies with different types of peptides moved in this direction, providing promising results in murine models of disease and opening new perspectives for peptide applications in the treatment of high-grade brain tumors.

## 1. Introduction

Astrocytomas are the most common primary glial tumors of the central nervous system (CNS). In 2016, the World Health Organization (WHO) revised the 2007 CNS tumors classification, discerning astrocytomas into circumscribed (WHO grade I) and diffuse (WHO grades II-IV) subtypes based on their histology and molecular parameters. The first subtype includes benign astrocytic tumors, such as pilocytic astrocytomas, which are usually treatable with complete surgical resection. Conversely, the second group includes those gliomas that are more difficult to treat because of their heterogeneity and invasive growth. These include diffuse astrocytoma (grade II), anaplastic astrocytoma (AA, grade III), and glioblastoma (GBM, grade IV) [1,2]. Depending on the presence or absence of isocitrate dehydrogenase 1 and 2 (*IDH1* and *IDH2*) gene mutations, this last group of tumors can be subclassified into IDH-mutant and IDH-wildtype categories, of which the first type is associated with better prognosis [3,4].

AA and GBM are two high-grade tumors. They are characterized by poor prognosis with a median survival of about 2–3 years and 12–15 months, respectively. Furthermore, they present with neurodegeneration, invasiveness, cytological pleomorphism, and increased mitotic activity. GBM exhibits also microvascular proliferation, necrosis, or both [5,6]. Patients show a wide range of symptoms, which depend on the brain area affected by the tumor. The most common signs are seizures, fatigue, headache and cognitive and motor disabilities. They negatively influence patients’ quality of life and can be mainly caused by the tumor itself or they can partially occur as side effects of the therapies [7,8].

The current treatment for AA and GBM consists of maximal surgical tumor resection, followed by chemotherapy with Temozolomide (TMZ) and focal radiotherapy. Furthermore, inpatient exercise rehabilitation programs after tumor resection were reported to have an important role to significantly improve neurocognitive and motor functions, which contribute to enhance the patients’ quality of life [9,10]. It is yet to mention that, even if currently available therapeutic protocols may be successful at the beginning, these types of tumor are highly drug-resistant and recurrence occurs in almost all cases [11]. These harmful features could be explained, at least partially, by the poor penetration of chemotherapeutics into brain tumor tissues due to the presence of the blood–brain barrier (BBB), with the consequent reduction of their efficacy. The BBB physiologically separates the brain parenchyma from the vasculature, protecting the CNS from circulating pathogens and toxins as well as regulating the transport of essential molecules. However, this dynamic and defensive interface limits also the passage of most hydrophilic drugs into the CNS, after systemic administration. Although high-grade brain tumors can damage the integrity of the BBB, this latter is not uniformly disrupted, thus preventing the achievement of effective drug concentrations in the tumor affected area [12]. On the other hand, the tumor itself is surrounded by a pathological barrier constituted by an abnormal extracellular matrix (ECM) that hampers the passage of drugs from the vasculature to the tumor parenchyma [13]. Besides, the failure of the treatments and the high recurrence rates of diffuse gliomas can also be ascribed to their high cellular heterogeneity. A number of studies have described, within the tumor, the presence of a subpopulation of cells, called glioma-stem-like cells (GSCs) or glioma-initiating cells (GICs), which are responsible for tumor generation, progression, invasion, angiogenesis, and recurrence. GICs are, in fact, characterized by self-renewal, indefinite proliferation and chemotherapy/radiotherapy resistance [14,15,16].

In view of all these considerations, the development of novel therapeutic approaches, capable to overcome both the BBB and the tumor barrier, and able to inhibit tumor growth by selectively targeting GICs emerges as an urgent need. In the last few years, several studies have been performed to realize a genetic and lineage classification of GICs in order to design targeted and personalized therapies [17,18,19]. It is clear that the achievement of these objectives would definitely maximize the therapeutic efficacy of anti-cancer drugs and reduce their side effects.

In this regard, the use of peptides for the treatment of a variety of diseases, including brain tumors, has been rapidly expanding. Some of these have already moved into Phase I/II clinical trials for the treatment of high-grade gliomas, showing promising results not only in terms of safety and tolerability, but also for their ability to reduce the tumor mass [20,21,22].

In this review, we provide an outline of the properties of different types of peptidic agents. Furthermore, we explore potential molecular targets for the treatment of high-grade astrocytomas using anti-tumor peptide therapeutics as well as peptide carriers able either to deliver anti-cancer molecules through cell and tissue barriers or to target tumor-specific receptors [23].

## 2. Peptides as Tools for Therapies

Peptides are a novel class of compounds that can be used for the treatment of a wide range of pathological conditions, such as infections, diabetes, cardiovascular diseases, neurodegenerative disorders, and cancer. They are low molecular weight molecules, usually 10–50 amino acids long. The advantages of peptides are several-fold and include ease of synthesis, high specificity and activity, and low production cost. Furthermore, in the case of cancer, peptides result less immunogenic than recombinant monoclonal antibodies already used to target tumor cells with anti-cancer drugs [24]. Since peptides do not accumulate in tissues and organs, they are also endowed with low toxicity, reducing the number of side effects compared to conventional chemotherapeutic agents [25,26]. Besides, peptides can exhibit some pitfalls that have been recently tackled with various strategies in order to achieve optimal performance and accelerate clinical development. For example, due to their hydrophilic nature, they show low membrane permeability, a feature that can reduce their efficacy when the targets are intracellular. A way to solve this problem was found in conjugating bioactive peptides with cell-penetrating peptides (CPPs). CPPs are a family of short amino acid sequences (5-30 residues) able to pass through tissue and cell membranes via energy-dependent or independent mechanisms without interacting with specific receptors. They can also convey into cells numerous biologically active conjugates (cargoes) other than peptides, including nucleic acids, small drugs, and proteins. Thus, they can be used to improve the delivery to tumor cells of different types of anti-cancer pharmacological agents [27].

In vivo, peptides have poor stability due to their susceptibility to degradation by serum proteases. This short half-life prevents the development of drug resistance and increases their safety, though it may also reduce their efficacy. This constraint can be prevented by chemical modifications of the peptide sequence, for instance by integration of D-amino acids, cyclization, use of unnatural amino acids that are uncleavable by endogenous proteases, and blocking the access to the N- and C- terminal fragments [28]. Another drawback of peptides concerns their poor oral bioavailability. This problem can be overcome by combining the administration of biologically active peptides with adjuvant(s) that allow to enhance the intestinal absorption and/or inhibit the enzymatic digestive process, thereby increasing their pharmacokinetic properties [29].

In the case of brain tumor treatments, peptides can be employed in several ways, depending on their specific properties. According to their mechanism of action, they can be classified into two major groups. The first class includes peptides that are characterized by a direct mode of action. Some of these molecules have an intrinsic anti-cancer activity, so that they can be used as therapeutics, promoting the reduction of tumor growth by a variety of mechanisms. For instance, anti-microbial peptides (AMPs) are short cationic and hydrophobic molecules that play a cytotoxic action by targeting the negative charges of cancer cell membranes and inducing cell death through apoptosis or necrosis [30,31]. Other peptides of this group act by inhibiting angiogenesis, cell cycle, specific signal transduction pathways (e.g., mitogen-activated protein kinase (MAPK) pathways) or transcription factors (e.g., c-Myc) [11,26]. This first set of peptides includes also those that can be used as drug delivery systems, i.e., CPPs, for their ability to translocate through cell and tissue membranes, as well as tumor-targeting peptides (TTPs). TTPs, also known as homing peptides, target specific markers that are expressed only by tumor cells, vasculature, and microenvironment. They can be used to deliver chemotherapeutics or cytotoxic peptides specifically to cancerous cells, without affecting normal tissues [26,28].

In the second group are peptide vaccines, which are characterized by an indirect mode of action. Cancer vaccines can be considered as active immunotherapies because they are based on the administration of tumor-associated antigens, which are specifically expressed in cancer tissues. The aim is to stimulate the immune system to react against the tumor [32]. In this article, we will not consider peptide vaccines, and we refer interested readers to other recent reviews on this topic [33,34,35].

## 3. Molecular Targets for Peptide-Based Treatments of Astrocytomas

Advancements in the comprehension of molecular, cellular and genetic anomalies of various tumors have enabled the development of the “Atlas of Genetics and Cytogenetics in Oncology and Haematology”, a database listing several genes and proteins that are relevant for different types of cancer, including diffuse glioma. Some of these have been investigated as potential targets for small molecules or peptide vaccines, while others were specifically addressed with peptidic agents [36,37,38,39]. Peptidic molecules can be used both as monotherapy as well as in combination with standard anti-cancer drugs. Besides, they can be exploited as medicaments, but also as delivery systems to transport chemotherapeutics into glioma cells or into the vasculature by targeting tissue-specific molecular markers.

Because most of the pathways that are altered in astrocytomas are primarily located within GICs, the main goal for the future is to develop strategies that specifically target this cellular subpopulation. Although several treatments conceived on this principle are still in the preclinical phase, there are solid promises for clinically relevant success.

Here below, we report some detailed examples of glioma targets that can be specifically addressed by peptide-based drugs (Table 1).

### 3.1. Molecular Targets on Glioma Cells/GICs

#### 3.1.1. CXCR4/CXCL12

In high-grade astrocytomas, such as GBM, an increased expression of the C-X-C chemokine receptor type 4 (CXCR4) has been reported. This can transduce growth signals in response to its protein ligand CXCL12. Clinically, CXCR4 expression levels in GBM correlate with increased tumor grade, aggressiveness and, consequentially, with a poor prognosis [48]. A study by Schulte and colleagues, demonstrated that not all cells of the tumor are CXCR4 positive, but its overexpression can be found in the GIC subpopulation, which becomes responsible for tumor invasion and metastasis [49,50]. The CXCR4-CXCL12 axis prompts the activation of numerous effectors, including extracellular-signal-regulated kinase 1/2 (ERK1/2), MAPK, protein kinase B (Akt), and Nuclear Factor Kappa B (NF-κB). In turn, this latter can induce the expression of several target genes involved in cellular proliferation and invasiveness [48]. Furthermore, it has been observed that CXCL12 is highly expressed by the endothelium, while CXCR4 is mostly concentrated on GICs surrounding the neovessels, suggesting a role of this signaling pathway in promoting angiogenic processes by directly inducing endothelial cell migration [51].

**Figure 1:**
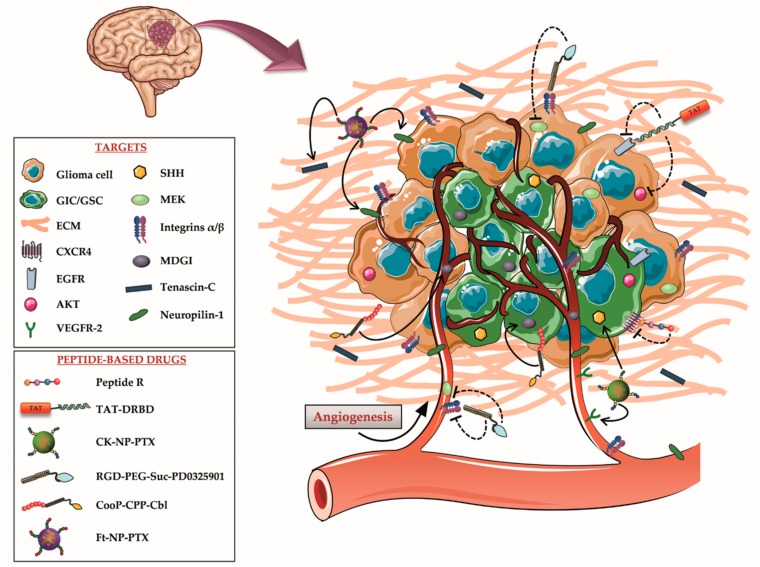
Schematic drawing of high-grade astrocytoma structure, microenvironment, and molecular targets. In the last few years, many key signaling pathways for astrocytoma development and invasiveness have been identified on different components of the tumor mass, including glioma-initiating cells (GICs), glioma cells, extracellular matrix (ECM) and vasculature. Several peptide therapeutics and peptide-based drugs, addressing these targets, have been subsequently designed to maximize the therapeutic efficacy of the treatments and reduce their side effects.

These observations indicate that the CXCR4-CXCL12 pathway mediates survival and self-renewal in GICs with high selectivity, emerging as an attractive target for glioma-directed therapies. The CXCR4 antagonist that was most widely tested in clinical trials to reduce the growth of GBM, is the small molecule Plerixafor [52]. Yet, its lack of receptor specificity is known to cause several adverse effects [53]. Thus, to overcome this issue, Mercurio and colleagues recently developed and evaluated, in an intracranial GBM mouse model, the direct therapeutic potential of the cyclic peptide R (RACRFFC), a synthetic and specific CXCR4 antagonist (Figure 1). Following chronic intraperitoneal administration of this peptide, researchers detected a decreased tumor cell density and dissemination in other brain areas; the generation of an unfavorable microenvironment for tumor cells; and a reduced tumor vasculature. The resulting data was comparable with the effects of Plerixafor, with the advantage of peptide R specificity for CXCR4, thereby encouraging to further explore its therapeutic potential, alone or in combination with standard therapies [40,54].

#### 3.1.2. EGFR/AKT

Epidermal growth factor (EGF) receptor (EGFR) is a transmembrane receptor tyrosine kinase involved in a wide variety of cellular processes and cancers, including GBM. EGFR is activated by a set of ligands, including EGF and transforming growth factor-alpha (TGF-α), which trigger its dimerization. The latter stimulates the autophosphorylation of the EGFR intracellular tyrosine kinase domain, leading to activation of numerous downstream signaling pathways, such as the phosphatidylinositol-3 kinase (PI3K)/AKT/rapamycin-sensitive mTOR-complex (mTOR) cascade. This signal transduction pathway is involved in various cellular functions, including cell cycle progression, differentiation, migration, and survival [55,56]. AKT is a serine/threonine kinase that, once activated, regulates cell cycle and pro-apoptotic/anti-apoptotic factors that promote cell survival, self-renewal and proliferation. Furthermore, it mediates also mechanisms of drug resistance, through NF-κB stimulation [55].

It has been described that approximately half of the patients affected by GBM overexpresses EGFR, and 20–30% of them expresses the mutant truncated variant EGFRvIII. The latter is constitutively active and promotes persistent intracellular signaling, with consequent tumor growth, survival, invasion, and angiogenesis [57]. Interestingly, the phosphatase and tensin homolog (PTEN), the main suppressor of AKT, is often inactive in GBM due to gene mutation or methylation. This situation leads to high levels of phosphorylated AKT in 70% of glioma cases and correlates with poor outcomes [58].

All these conditions cause a continuous activation of the PI3K/AKT/mTOR pathway, an event that has been described also in GICs, and this contributes to GBM progression and drug resistance [59]. Several potential treatments against EGFR or EGFRvIII, including monoclonal antibodies and vaccines, are currently in development or in clinical trials. However, their efficacy is limited, due to glioma cell resistance mechanisms that overcome the inhibition through increased expression of other growth factors. This suggests that the design of a multiple target approach may increase the inhibitory effect on cell invasion and angiogenesis, thereby being more effective [60,61]. Among others, EGFR and AKT emerge as possible molecular targets for combinatorial therapeutic interventions. Related to this point, it should be mentioned that the group of Steven F. Dowdy tested the potential of a peptide-based therapy to kill GBM tumor cells in vivo, by targeting the EGFR and AKT oncogenes. More specifically, they generated an efficient short interfering RNA (siRNA) delivery approach by conjugating a double-stranded RNA-binding domain (DRBD) with a CPP, the *trans*-activator of transcription (TAT) protein from HIV-1, since the large size and negative charges of siRNA hinder their cellular translocation. This system, named TAT-DRBD, was used to deliver EGFR and AKT siRNAs into an intracranial GBM mouse model to induce synthetic lethal RNA interference responses (Figure 1). This approach drove a reduction in the tumor volume, extensive apoptotic cells, and a significant increase in the median survival of treated mice, exhibiting a great potential for GBM targeted therapy [41,42].

### 3.2. Molecular Targets on Glioma Cells/GICs and Blood Vessels

#### 3.2.1. VEGFR-2 and Human Sonic Hedgehog

Extensive vascularization is a typical feature of GBM and one of the main causes of its poor prognosis. The reason for this can be found in the fact that angiogenesis is critically involved in tumor development, growth, and invasiveness. Anti-angiogenic therapeutic strategies against molecular targets placed on tumor vascular endothelial cells thus appear as efficient approaches to counteract glioma progression. In many cancers, including GBM, the effectiveness of such treatments may be, however, compromised by the existence of another vascular network structure, complementary to the tumor vasculature, and consisting of vasculogenic mimicry (VM) channels [43]. These channels are particular blood vessels formed by tumor cells, rather than endothelial cells, reconverted to an embryonic-like phenotype. They are able to mimic endothelial functions or act as common tumor cells [62,63]. Furthermore, VM channels are highly resistant to conventional anti-angiogenic therapies, causing tumor recurrence [43]. This amount of evidence suggests that targeting VM channels, besides tumor neovasculature, may be a possible approach to improve the efficacy of glioma treatments. Vascular endothelial growth factor receptor-2 (VEGFR-2), a key mediator of angiogenic process, self-renewal and tumorigenicity, emerges as a promising therapeutic target, being highly expressed on both traditional tumor vasculature and VM channels [62]. This evidence has prompted a number of clinical trials with VEGFR-2 inhibitors that allowed to target both types of vasculature. However, anti-angiogenic treatments resulted to exhibit only transitory benefits, followed by an increased risk of drug-resistance, glioma metastasis, and recurrence that culminate in limited patient survival [64]. The first reason for this failure lies in the hostile hypoxic tumor microenvironment engendered by the anti-angiogenic therapy, from which tumor cells try to escape generating distant metastasis. On the other hand, GICs become more hypoxia tolerant and resistant, contributing to glioma recurrence by self-renewal and to drug-resistance [46,65].

These findings reveal that a combinatorial therapy, simultaneously targeting neo-angiogenesis and glioma cells, might represent an efficacious approach, since not only it would hinder oxygen and nutrients supply, but also kill tumor cells.

Mounting evidence attributes GIC proliferation and resistance to deregulated pathways, such as Hedgehog (HH) signaling, which may be an effective target to improve GBM therapies. This signaling begins with the binding of HH ligands, more frequently sonic hedgehog (SHH), to the transmembrane receptor Patched, which initiates an intracellular signaling cascade that results in the activation of the family of Gli transcription factors. The upregulation of the HH/Gli1 pathway is associated with worse prognosis in GBM patients, because it is implicated in the regulation of cellular proliferation, survival, invasion, and angiogenesis [66,67].

Based on these observations, Feng and colleagues developed Paclitaxel (PTX)-loaded nanoparticles (CK-NP-PTX) coated with a previously tested TTP, named CK peptide. This was composed of a VEGFR-2 targeting peptide (K237) bound, via a GYG linker, with a SHH targeting peptide (CVNHPAFAC-NH_2_), isolated from phage display libraries (Figure 1) [43,68].

This drug delivery system was designed with the aim of increasing the therapeutic efficacy of glioma treatments, by simultaneously targeting the chemotherapeutic PTX to VM channels, tumor neovasculature, and GICs. To test the system, an intracranial glioma mouse model was injected intravenously with CK-NP-PTX. Researchers observed a selective accumulation of the compound around the vasculature as well as in the tumor parenchyma. This distribution determined a strong VM channels destruction, a significant apoptosis of glioma cells and an increase in medium survival time in treated mice when compared to controls [43].

#### 3.2.2. MEK/ERK and Integrins

The MAPK kinase (MEK)/extracellular signal-regulated kinase (ERK) signaling pathway has been identified as a commonly dysregulated pathway in several cancers, including GBM. This cascade starts with the binding of a ligand to a transmembrane receptor tyrosine kinase and culminates with the phosphorylation through MEK of the final MAPK ERK. This latter translocates to the nucleus where it activates numerous transcription factors (e.g., c-Jun, c-Myc, and NF-κB) involved in the regulation of a large variety of processes including cell proliferation, differentiation, migration, and apoptosis [69]. Li and collaborators found, in a GBM mouse model, the involvement of this pathway in tumor development and invasiveness, suggesting MEK1/2 as promising therapeutic targets [70].

Integrins are a big family of cell adhesion transmembrane receptors composed of two associated α and β subunits, which directly bind several components of the ECM providing the adhesion required by tumor cells for their motility and invasion. Although integrins do not act as oncogenes, they cooperate with them or with receptor tyrosine kinases to increase tumorigenesis [71]. Some types of integrins, such as α_v_β_3,_ are overexpressed in GBM, both on the surface of tumor cells as well as on angiogenic vessels. They contribute to angiogenesis and correlate with worse prognosis [44,72,73,74]. These observations suggest that therapeutic strategies based on integrin antagonists may be valuable tools to modulate GBM growth and infiltration, slowing down the progression of the disease. The first integrin antagonist to move into clinical trials was Cilengitide, a cyclic pentapeptide containing the RGD (arginine/glycine/aspartic acid) motif, which makes it a selective antagonist of α_v_β_3_ and α_v_β_5_ integrins, preventing their interaction with ECM ligands. However, the promising results obtained in pre-clinical studies and in earlier clinical trials, were disconfirmed from the CENTRIC phase III trial that produced no survival benefit in treated patients when compared to controls [75].

Another possible use for RGD peptides is to exploit them as TTPs, in order to increase the specificity of anti-cancer drugs and deliver them directly to the tumor. In view of all these observations, Hou and colleagues developed a system to combine tumor-targeting drug delivery with two therapeutic agents characterized by distinct mechanisms. More specifically, they designed a new peptide-based drug, named RGD-PEG-Suc-PD0325901, by conjugating the MEK1/2 inhibitor PD0325901, through a mini-PEG linker, with an α_v_β_3_ integrin antagonist RGD peptide. The co-delivery of PD0325901 and the RGD peptide allowed synergetic effects with inhibition of the ERK pathway, which is overactivated in GBM cells, and disruption of angiogenic signals in GBM tissue as well as inhibition of cell migration. Moreover, the RGD peptide permitted to deliver the MEK1/2 inhibitor to tumor cells and to the vasculature by integrin-targeted delivery, preventing off-target effects on healthy tissues (Figure 1). To evaluate the efficacy of the system, a GBM subcutaneous xenograft mouse model was intravenously injected with RGD-PEG-Suc-PD0325901. This compound determined a significant inhibition of cancerous cell proliferation, associated with a reduction in tumor size [44].

#### 3.2.3. MDGI

Another possible therapeutic approach to hinder brain tumor recurrence, targeting both invasive GICs and tumor vessels, consists in targeting the mammary-derived growth inhibitor (MDGI). MDGI is a fatty acid-binding protein whose role in tumorigenesis is rather controversial and seems to vary in a cancer type-dependent manner. It can be linked to tumor-suppressor properties, e.g., in breast cancer [76], but also to tumor-promoting functions, such as in GBM. In this latter, MDGI was shown to be highly expressed both in GICs, in which it regulates cell viability, invasive growth, and lysosomal integrity, as well as in the blood vasculature endothelium [45]. High MDGI expression correlates with poor outcomes [77], thereby emerging as an ideal target for delivering anti-tumor drugs to glioma.

To this end, the group of Pirjo Laakkonen identified a novel synthetic tumor homing peptide, named CooP (ACGLSGLGVA), which specifically targets invasive tumor cells and the vasculature by binding to MDGI. They subsequently investigated the potential of CooP-targeted therapy to treat high-grade brain tumors. Thus, they developed a peptide-drug conjugate, named CooP-CPP-Cbl, in which the CooP peptide was covalently conjugated with the chemotherapeutic agent clorambucil (Cbl) and a CPP derived from the N-terminal part of the tumor suppressor protein p14ARF (MVRRFLVTLRIRRACGPPRVRV-NH2), to permit cellular internalization (Figure 1). To assess the efficacy, an intracranial tumor mouse model was intravenously injected with the peptide-based drug. This treatment successfully prolonged the mouse lifespan and reduced the quantity of invasive tumor cells when compared to controls [45]. Subsequently, another group of researchers used the same tumor homing peptide to functionalized nanoparticles loaded with the chemotherapeutic drug PTX, enhancing anti-tumor efficacy. They injected glioma-bearing mice with the nano-vector CooP-NP-PTX, founding a longer survival when compared to mice treated with untargeted NP-PTX [46].

### 3.3. Molecular Targets on Glioma Cells, Blood Vessels and Extracellular Matrix

#### Tenascin-C and Neuropilin-1

As mentioned in the previous paragraphs, the most promising strategy to treat high-grade astrocytomas seems to be the simultaneous targeting of neovasculature/VM channels as well as glioma cells or GICs. Yet, an additional aspect that should be considered attentively is the poor penetration of most drugs in the tumor mass, due to the presence of a pathologic barrier constituted by ECM. The occurrence of this event is one of the causes of GBM recurrence [47]. The ECM is a network of extracellular macromolecules that provides structural and biochemical support to the surrounding cells. During embryonic development and in physiological conditions, ECM results to be highly organized, but it becomes deregulated in various types of cancer, including high-grade astrocytomas. Glioma cells themselves up-regulate the secretion of matrix components, leading to the condensation of ECM, and this might prevent the passage of molecules and drugs from the circulation to the tumor parenchyma. Furthermore, it has been shown that alterations in ECM composition may affect cancer progression by generating a tumorigenic microenvironment, which promotes metastasis formation and facilitates angiogenic processes [13,78]. Thus, the identification of anti-cancer molecules that target glioma cells, but also ECM markers, may be an attractive approach to improve drug penetration into the tumor mass.

Among ECM components, tenascin-C (TN-C) is a hexameric glycoprotein mainly expressed by neural and endothelial cells during embryogenesis. It is downregulated in adult healthy brains, but it becomes overexpressed in about 90% of GBMs. Tumor cells are the main source of TN-C release, and the intensity of its expression correlates with glioma grade and outcomes. TN-C is able to bind other ECM proteins as well as integrin receptors, thus influencing a number of cellular processes, such as cell migration, angiogenesis, and proliferation [79]. For all these reasons, TN-C may be considered as an interesting target for glioma therapy.

Neuropilin-1 (NRP-1) is a multifunctional non-tyrosine kinase co-receptor expressed in many tissues, which binds a number of factors, including VEGF-A, Hedgehogs, TGFβ and EGF. It was shown to be highly expressed in GBM cells and neo-vasculature, where it regulates glioma growth, progression, and recurrence. The expression of NRP-1 correlates with glioma grade and poor patient prognosis [80,81,82].

In a recent study, Kang and collaborators developed a synergistic nanosystem constituted by PTX-containing nanoparticles coated with the synthetic Ft peptide (Ft-NP-PTX), which contains two sequences (i.e., FHK (FHKHKSPALSPV) and tLyp-1 (CGNKRTR) coupled via a cysteine) to simultaneously target TN-C and NRP-1, respectively. This system was designed to specifically circumvent the ECM barrier by targeting the glioma-related matrix component TN-C, and to concurrently achieve deep penetration in the glioma parenchyma, mediated by the over-expression of NRP-1 in glioma cells and vasculature (Figure 1). Mice bearing intracranial glioma were systemically injected with Ft-NP-PTX. A significant increase in median survival was detected when compared to untreated mice [47]. These results emphasize the importance of promoting the development of multiple-targeting therapies to improve the effectiveness of gliomas treatments.

## 4. Concluding Remarks

Over the last years, considerable progress has been made with regard to the development of pharmacological treatments for high-grade astrocytomas. Nonetheless, the prognosis of these types of cancer has not significantly improved and they remain the most aggressive and lethal tumors of the CNS. Even after total or subtotal surgical eradication of the tumor, followed by radiotherapy and concomitant adjuvant chemotherapy, the median survival does not exceed 3 years. There are many reasons for such a failure, including the critical brain localization and the lack of defined margins, which might hinder the total resection of the tumor mass. This event may contribute to its recurrence. Such disappointing results can be explained also with the structural complexity of malignant gliomas. In fact, it is well known that they are characterized by a great cellular heterogeneity and, within the tumor tissue, different sub-populations of poorly differentiated cells co-exist. Among these, GICs are the main responsible for tumor invasiveness and recurrence. These tumors are also supported by a complex network of blood vessels, including standard vasculature, which is constituted by endothelial cells, and VM channels, formed by glioma cells that are able to act as both endothelial and tumor cells. Finally, the complexity of high-grade gliomas can also be ascribed to the presence of an ECM-anomalous barrier that surrounds the tumor. This latter, together with the BBB, hampers the access of drug molecules to the tumor parenchyma.

All these aspects make these types of cancer resistant to any kind of therapy. Furthermore, most chemotherapeutic agents are not selective for cancer cells, but they also damage healthy tissues, thus leading to diffuse adverse effects.

In recent years, a significant number of key signaling molecules have been identified in many pathways, specifically altered in malignant gliomas, allowing the advent of more effective targeted and personalized therapies. In this framework, peptides have emerged as a novel class of etiology-based anti-cancer therapeutics that can be used as (i) pharmacologically active agents or (ii) carriers, either to facilitate the translocation of chemotherapeutics through brain, tumor, and cellular barriers, or to target tumor-specific molecular markers. This approach should make the therapy more specific, i.e., more effective and characterized by fewer side effects.

Another aspect to consider carefully is that single-target approaches have not resulted in improved prognosis for patients affected by malignant gliomas. Better outcomes may result from combining multi-target strategies. For instance, peptide-based therapies can be designed to simultaneously target multiple effectors either on the same pathway or involved in different mechanisms and tumor compartments. To achieve this goal, it is necessary to target diverse markers on (i) ECM components, to enable the achievement of effective drug concentrations in the tumor mass; (ii) standard vasculature and VM channels, to remove nutrients and oxygen supply to the tumor; and (iii) glioma cells/GICs to kill those cells that become resistant to the hypoxic microenvironment and are responsible of metastasis formation. The successful elaboration of these approaches may enable, in the future, the development of effective personalized molecular therapies for high-grade astrocytomas.

## Figures and Tables

**Table 1:** Main anti-cancer peptides acting on molecular targets that have been tested in preclinical studies.

Molecular Target	Target Localization	Mediated Function	*Peptide*-Cargo	Type of Peptide	Ref.
CXCR4	GICs	Survival Self-renewal Angiogenesis	*Peptide R*	Peptide therapeutic	[40]
EGFR; AKT	GICs/glioma cells	Proliferation Migration Survival Self-renewal Drug resistance	*TAT*-DRBD	CPP	[41,42]
VEGFR-2; SHH	Neo-vasculature/VM channels; GICs	Angiogenesis Proliferation Survival Migration	*CK*-NP-PTX	TTP	[43]
MEK/ERK; Integrins	Glioma cells/neo-vasculature	Proliferation Differentiation Invasiveness; Migration Angiogenesis	*RGD*-PEG-Suc-PD0325901	TTP	[44]
MDGI	GICs/neo-vasculature	Cell viability Invasive growth Angiogenesis	*CooP-CPP*-Cbl; *Coop*-NP-PTX	TTP-CPP TTP	[45,46]
Tenascin-C; Neuropilin-1	ECM; Glioma cells/neo-vasculature	Migration Angiogenesis; Growth Progression Recurrence	*Ft*-NP-PTX	TTP-CPP	[47]
